# Knowledge, Attitudes, and Practice of Pelvic Floor Muscle Training in People With Spinal Cord Injury: A Cross-Sectional Survey

**DOI:** 10.3389/fresc.2022.893038

**Published:** 2022-06-14

**Authors:** Maya Sato-Klemm, Alison M. M. Williams, W. Ben Mortenson, Tania Lam

**Affiliations:** ^1^School of Kinesiology, University of British Columbia, Vancouver, BC, Canada; ^2^International Collaboration on Repair Discoveries (ICORD), Vancouver Coastal Health Research Institute, Vancouver, BC, Canada; ^3^Occupational Science and Occupational Therapy Department, University of British Columbia, Vancouver, BC, Canada; ^4^Rehabilitation Research Program, GF Strong Rehabilitation Centre, Vancouver, BC, Canada

**Keywords:** pelvic floor (MESH unique ID = D017773), spinal cord injuries (MeSH), rehabilitation, exercise, knowledge, attitude, practice

## Abstract

**Background:**

There is emerging evidence that pelvic floor muscle training (PFMT) may be useful for treating some urogenital conditions in people with spinal cord injury (SCI). Future clinical investigations would benefit from understanding the extent to which people with SCI are aware of and practicing PFMT, and their attitude toward this therapy.

**Objective:**

The goal of this study was to assess the knowledge, attitudes, and practices related to PFMT among people with SCI.

**Methods:**

We distributed an internet survey internationally *via* SCI related organizations for 2 months. We used descriptive statistics to summarize each survey item, and Chi-square and Mann-Whitney U tests to explore the differences in results between sexes and level of motor-function.

**Results:**

Complete data from 153 respondents were analyzed. Sixty-two percent of respondents were female and 71% reported having complete paralysis. More than half of respondents reported being aware of PFMT (63%); more females than males reported knowledge of PFMT (*p* = 0.010). Females (*p* = 0.052) and people with partial paralysis (*p* = 0.008) reported a stronger belief that they would benefit from PFMT. Few people with SCI had practiced PFMT (20%), and of those who practiced, most of them had SCI resulting in partial paralysis (*p* = 0.023).

**Conclusions:**

While people with SCI may be aware of and have favorable attitudes toward PFMT, few had practiced PFMT and there were notable differences in attitudes toward PFMT depending on the sex and level of motor function of the respondents.

## Introduction

People with spinal cord injury (SCI) face multiple health complications to many of the major physiological systems. Impairments in urinary function, secondary to denervation of pelvic structures including the bladder, urethral sphincters, and pelvic floor muscles are a common health concern, with ~80% of people with SCI experiencing neurogenic lower urinary tract dysfunction (1, 2). As the physiological and psychosocial consequences of chronic urinary impairments have serious implications on overall quality of life, it is no surprise that recovery of bladder function is of utmost priority for people with SCI (3, 4).

In able-bodied populations, pelvic floor muscle training (PFMT) is used to treat conditions such as urinary incontinence and sexual dysfunction in both males and females (5–7). This type of training involves practicing various combinations of contracting and relaxing the pelvic floor muscles in order to improve the strength, endurance, and coordination of this muscle group (8, 9). With pelvic floor muscle activation, the muscles squeeze around the urethra and lift upward, while reflexively inhibiting detrusor activity. These mechanisms can be refined through PFMT to improve urinary incontinence and other pelvic disorders (1, 2, 8, 9).

There is some preliminary evidence that PFMT may be used to improve neurogenic lower urinary tract dysfunction and male sexual health in people with SCI (10–12). After engaging in a PFMT program, individuals with SCI have reported less incontinence, fewer episodes of neurogenic detrusor activity, and reduced severity of erectile dysfunction (10–12). However, studies to-date have included relatively small sample sizes and only people with motor-incomplete SCI. There is evidence that despite their clinical diagnosis, people with complete paralysis after SCI are able to voluntarily activate some muscles below their level of injury (13–17), including the pelvic floor muscles (18). Thus, while people with motor-incomplete SCI may be more likely candidates for PFMT, there may also be opportunities to introduce PFMT to the motor-complete SCI population.

PFMT is not commonly prescribed to people with SCI, and the perspectives of this population toward to PFMT remains unknown. Understanding a population's knowledge, attitudes, and practices (KAP) about an intervention can reveal behavior intention toward health outcomes (19, 20). Previous studies that have assessed KAP of PFMT most often included able-bodied pregnant or post-partum females (21–24). These studies demonstrated that many participants were unaware of the benefits of PFMT and were consequently not participating in a PFMT program. Interestingly, despite having minimal knowledge of PFMT, respondents had positive attitudes toward learning more about this therapy and engaging in a PFMT program (21–24). The results from KAP surveys may therefore reveal opportunities to broaden a population's awareness and uptake of a given therapy or exercise program (19).

Although the benefits of PFMT have been predominantly explored in females (5, 8), there are clinical studies demonstrating that PFMT can also treat incontinence and sexual dysfunction in males (7, 25, 26). To date, there is little information on the KAP of males relating to PFMT in either clinical or able-bodied populations. As the majority of the SCI population is male (27, 28), it is also relevant to explore KAP within the context of sex to identify possible differences between the experience and perspectives of males and females.

Given the potential opportunities to explore the benefits of PFMT in the SCI population, and the importance of understanding perspectives on exercise program participation, the primary purpose of this study was to assess the KAP related to PFMT in the SCI population using an online questionnaire. A secondary aim was to explore differences in KAP in people with motor-incomplete vs. motor-complete SCI, and males vs. females with SCI. This information can be used to inform health practitioners and aid designers of PFMT programs specifically for the SCI population.

## Methods

Data for this study were collected using an online survey (29). Prior to distribution, we sought feedback on a draft version of the survey from a small group of knowledge-users, including two clinicians specializing in pelvic floor physiotherapy and sexual health, and one male and one female with SCI. We made minor adaptations based on their feedback. The University of British Columbia's Behavioral Research Ethics Board approved this study. We report survey findings according to the Checklist for Reporting Results of Online E-Surveys (CHERRIES) (30).

### Participants

Individuals were eligible to participate in the study if they reported sustaining a SCI due to a traumatic (e.g., motor vehicle accident, fall) or non-traumatic event (e.g., illness, infection) resulting in partial or complete paralysis. Additionally, participants had to be over 18 years of age, be able to provide informed consent, and have access to the internet. Participants were excluded from this study if they reported having any neurological condition other than a SCI or were unable to read and understand English.

### Survey Construction

A four-part cross-sectional survey (Data Sheet 1) was created using the Qualtrics Survey tool (Qualtrics Survey Platform, RRID:SCR_016728) to capture current knowledge, attitudes, and practice related to PFMT, following the guidelines for internet-based surveys (29). The survey had logic that presented only the necessary questions to the respondent (e.g., if a respondent indicated they have never done PFMT, they would not be asked for details about their program). As a result, the survey ranged from 1 to 5 items per page spanning 14–22 pages. After finishing each page, completeness checks were performed where the respondent was notified if they had not responded to a given question, giving them the opportunity to revisit that question, or leave it empty and proceed to the next page. Respondents were not able to go back and change their answers after a subsequent page was loaded.

### Part 1: Knowledge

We used Yes/No/Unsure multiple-choice questions to explore knowledge about PFMT. Additional fill-in-the blank style questions probed the respondents' knowledge of how they thought they could access PFMT and the purpose of PFMT.

Following the knowledge-themed questions, respondents were presented with a short education item about pelvic floor muscle anatomy and training (Data Sheet 1). We did this to provide basic information about PFMT to all respondents before they moved on to the next two parts of the survey.

### Part 2: Attitudes

Survey questions in this section asked respondents to rate their comfort in discussing PFMT with various individuals (e.g., a physician, a family member), how confident they were in their ability to contract their pelvic floor muscles, whether they wanted to learn more about PFMT, and whether they thought they could benefit from PFMT. Respondents used a 5-point Likert-type scale, ranging from strongly agree (5) to strongly disagree (1), to answer these questions.

### Part 3: Practice

We used Yes/No/Unsure multiple-choice questions to ask respondents if they had been informed about PFMT by a healthcare practitioner and if they had ever participated in PFMT. Respondents who indicated they had participated in PFMT were prompted to answer further questions about the training parameters of their program and whether they found it beneficial.

### Part 4: Demographics

At the end of the survey, we asked respondents to provide demographic details (age, sex, gender identity, chronicity, level of injury, severity of injury, location of residence, and highest level of education). In addition, we asked respondents if they could feel sensation or contract their muscles below their level of injury.

### Recruitment and Administration

The survey was promoted through national (e.g., Spinal Cord Injury BC) and international (e.g., NorCal SCI) SCI organizations. We provided organizations with a link to the online questionnaire that could be distributed *via* electronic newsletters or social media. Respondents had the ability to forward the link (i.e., informal snowball sampling). We offered a chance to be entered into a random draw for one of ten $50 gift cards as an incentive to participate. The survey was open for 2 months, between March and May, 2021.

### Data Analysis

We considered submissions to be invalid if the submission was not completed (i.e., the individual did not finish and submit the survey). To screen participants who likely did not have a SCI, submissions were considered invalid if a respondent selected multiple levels of spinal injury from the drop-down list (e.g., C7, T4, L2, and S1), or two levels of spinal injury with >2 levels between each (e.g., injury between T1–T11). Additionally, we considered submissions invalid if the survey completion time was under 1 min, if non-English answers were used (for fill in the blank responses) or if inconsistent answers were used (e.g., initially responding that they have never heard of PFMT, PFM, or Kegels, but then later indicating they are currently participating in a PFMT program).

We used descriptive statistics to summarize the demographic data and each item in the Knowledge, Attitudes, and Practice sections of the survey. We categorized respondents as having a motor-incomplete SCI (miSCI) if they reported being able to contract their muscles below their level of injury and as having a motor-complete SCI (mcSCI) if they reported not being able to contract any muscle below their level of injury. We categorized respondents as tetraplegic if they reported their injury as above T1 or paraplegic if their injury was located at or below T1. To explore the differences between male and female respondents and respondents with miSCI vs. mcSCI, we used Chi-square tests for independence to compare nominal data from the Knowledge and Practice sections and Mann-Whitney U tests to compare ordinal data from the Attitudes section of the survey. We performed *post-hoc* pairwise z-tests on significant results from the Chi-square tests with a Bonferroni corrected alpha level. All statistical analysis was completed using SPSS 27 (IBM, Armonk, NY) and statistical significance was evaluated at *p* < 0.05.

## Results

### Demographics

We received complete data from 153 respondents. The demographic characteristics of the respondents are presented in Table 1. The majority of respondents were female and respondents ranged in age from 21 to 79 years. Approximately one third of respondents reported having tetraplegia and just over a quarter of respondents were categorized as having mcSCI. Chronicity of SCI ranged from a few months to 62 years. Almost all respondents reported living in the United States or Canada, and the majority reported living in an urban setting and having achieved college-level education or higher.

**Table 1:** Respondents' demographic characteristics (*n* = 153).

**Characteristic**	***n* (%) or M ±SD**
**Sex**	
Female	95 (62.1)
**Age**	46.5 ± 14.3
**Years post-injury**	10.6 ± 13.9
**Cause of injury**	
Traumatic	127 (83.0)
**Level of injury**	
Cervical	39 (30.7)
Thoracic	53 (41.7)
Lumbar	32 (25.2)
Sacral	3 (2.4)
Non-traumatic	26 (17.0)
**Muscle function (self-report)**	
Motor-incomplete	109 (71.2)
**Sensory function (self-report)**	
Sensory-incomplete	110 (71.9)
**Country**	
Canada	35 (22.9)
United States of America	111 (72.5)
Other	7 (4.6)
**Location of residence**	
City	81 (52.9)
Suburban	50 (32.7)
Rural	22 (14.4)
**Level of education completed**	
Less than high school	2 (1.3)
High school	31 (20.3)
College or trade school	82 (53.6)
Post graduate degree	38 (24.8)

### Knowledge

**Figure 1:**
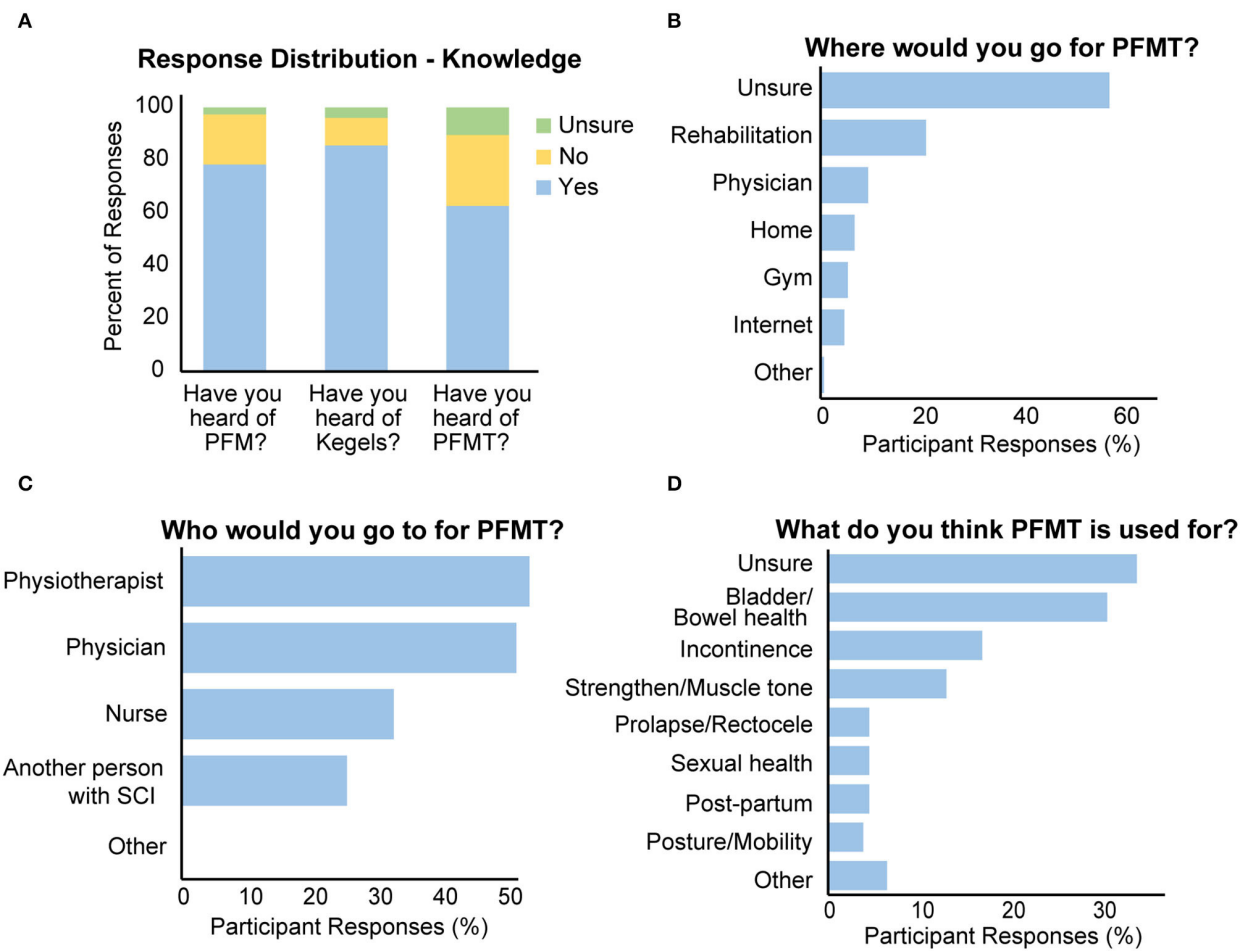
Results from the knowledge portion of the questionnaire. **(A–C)** Responses from knowledge question prompts. The title of the chart indicates which question was asked. **(D)** Results from the knowledge portion of the questionnaire.

Seventy-eight percent of respondents reported that they had heard of the pelvic floor muscles, or were familiar with the terms “Kegels” (86%) or “pelvic floor muscle training” (63%) (Figure 1A). More females than males reported having heard about the pelvic floor muscles or PFMT, but there was no difference between people with miSCI and mcSCI (Table 2).

**Table 2:** Knowledge results by sex and degree of motor function.

**Question**	**Response**	**Females *n* (%)**	**Males *n* (%)**	**miSCI *n* (%)**	**mcSCI *n* (%)**
Have you heard of PFM?	Yes	***82 (86)***	***38 (66)***	87 (80)	33 (75)
	No	***11 (** **12** **)***	***18 (** **31** **)***	18 (17)	11 (25)
	Unsure	2 (2)	2 (3)	4 (4)	0
		**χ^2^ = 9.427**, ***p*** **= 0.009**	χ^2^ = 2.898, *p* = 0.235
Have you heard of Kegels?	Yes	86 (91)	45 (78)	94 (86)	37 (84)
	No	6 (6)	10 (17)	10 (9)	6 (14)
	Unsure	3 (3)	3 (5)	5 (5)	1 (2)
		χ^2^ = 5.188, *p* = 0.075	χ^2^ = 1.042, *p* = 0.594
Have you heard of PFMT?	Yes	***68 (72)***	***28 (48)***	72 (66)	25 (55)
	No	21 (22)	20 (35)	26 (24)	15 (34)
	Unsure	***6 (** **6** **)***	***10 (** **17** **)***	11 (10)	5 (11)
		**χ^2^ = 9.286**, ***p*** **= 0.010**	χ^2^ = 1.936, *p* = 0.380

*Comparison of Knowledge between sex and motor-functions. Bolded values indicate significant differences. Bolded and italicized values indicate comparisons are significant through post-hoc testing*.

When asked about where one could access PFMT, the majority (58%) of respondents were unsure (Figure 1B). More people with mcSCI compared to those with miSCI [χ^2^ (1) = 7.726, *p* = 0.005] and more males than females [χ^2^ (1) = 15.398, *p* < 0.001] reported being unsure of where to go for PFMT.

When asked about whom they could approach for PFMT, the majority of respondents indicated that they would go to a physiotherapist (54%) or a physician (52%), and about a third indicated they would go to a nurse and a quarter indicated they would go to another person with SCI (Figure 1C). There were no significant differences between males and females or people with mcSCI and miSCI in the response pattern to this question.

When asked what they thought PFMT could be used for, 34% of respondents stated they were either unsure and 31% indicated general bladder/bowel health (Figure 1D). A smaller proportion of respondents specifically stated that PFMT could be used to treat incontinence (17%), strengthen the pelvic floor muscles (13%), treat prolapse (4.6%), or improve sexual health (4.6%).

### Attitude

**Figure 2:**
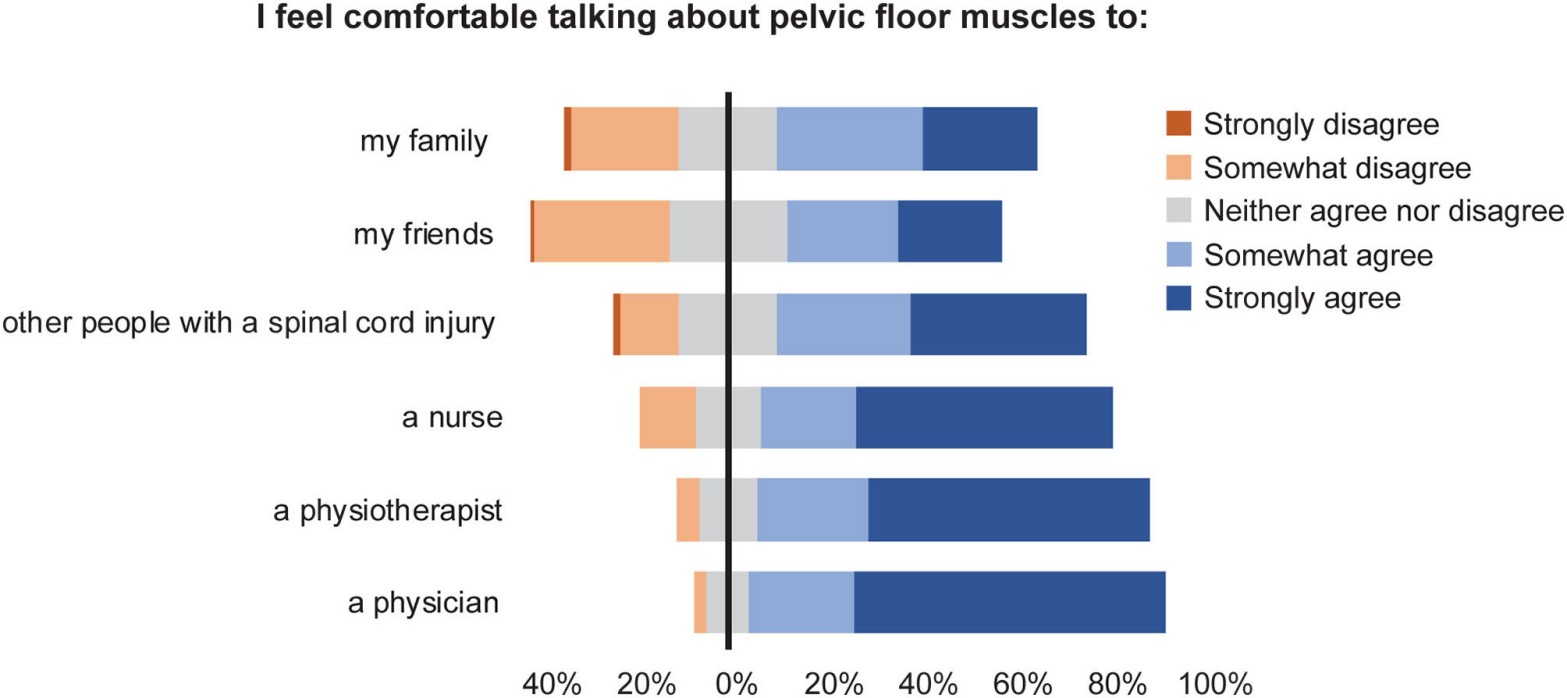
Responses to the question “I feel comfortable talking about pelvic floor muscles to _____” (Attitudes).

Most respondents agreed or strongly agreed that they would be comfortable talking about PFMT with a health care professional, including a physician (88%), a physiotherapist (83%), or a nurse (75%) (Figure 2). Respondents also indicated they would be comfortable talking to another person with a spinal cord injury (65%), a family member (55%), or a friend (46%) about PFMT. There were no differences between male or female respondents when considering who they would feel comfortable talking to about PFMT. When comparing mcSCI and miSCI respondents, those with mcSCI were more comfortable discussing PFMT with another person with SCI (U = 1,862, *p* = 0.024), but there were no other significant differences in response pattern.

**Figure 3:**
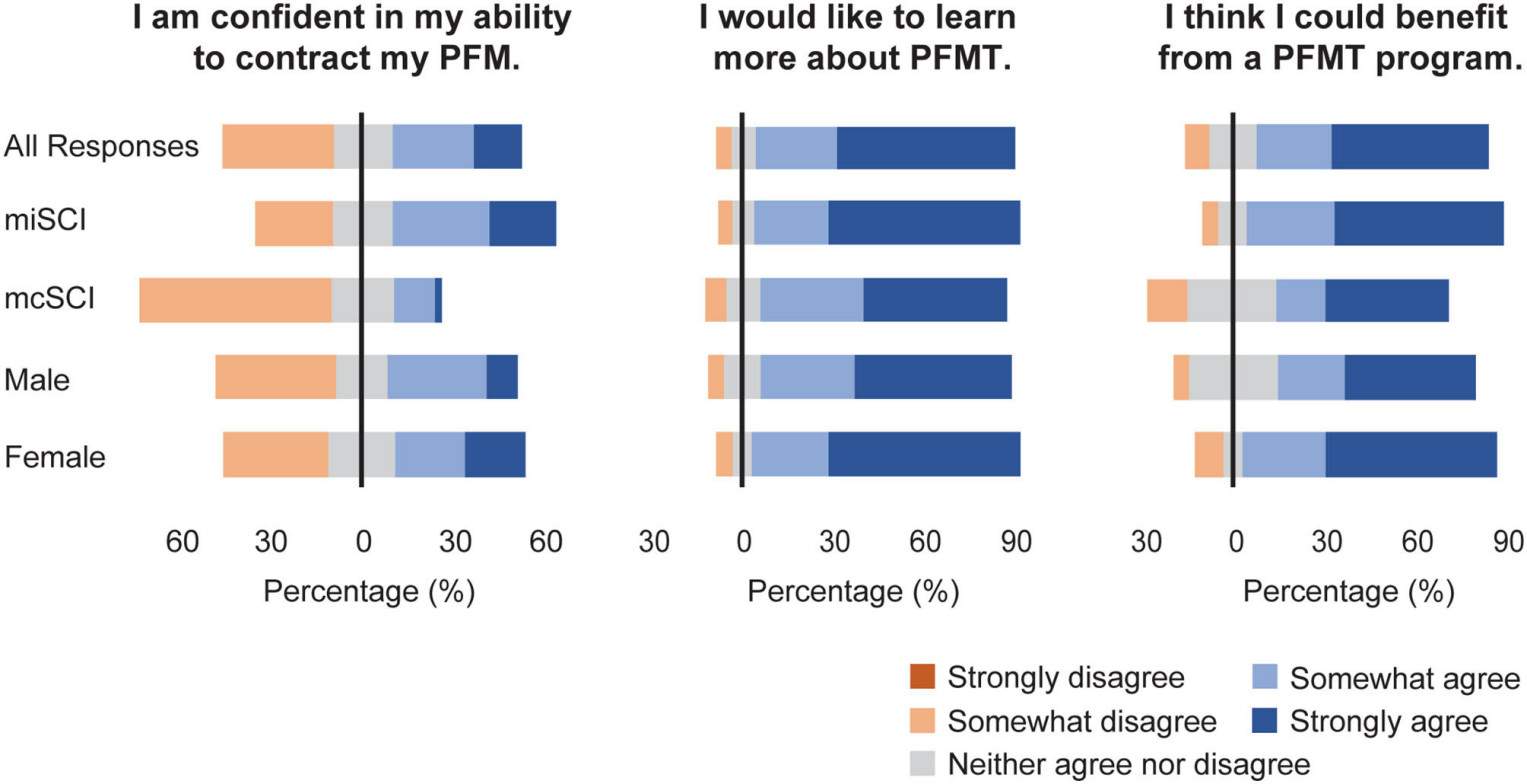
Attitudes to PFMT, reported for the complete sample and grouped by motor function and sex.

Forty-three percent of respondents agreed or strongly agreed with the statement that they were confident in their ability to contract their pelvic floor muscles, 86% indicated that they would like to learn more about PFMT, and 77% reported that they believed they would benefit from PFMT (Figure 3). Compared to respondents with mcSCI, those with miSCI were more confident in their ability to contract their pelvic floor muscles (U = 1245.0, *p* < 0.001) and believed they would benefit from PFMT (U = 1798.0, *p* = 0.008). There was a trend toward females feeling more strongly than males that they would benefit from PFMT (U = 2281.5, *p* = 0.052). There were no other differences in attitudes toward PFMT between males and females or miSCI vs. mcSCI.

### Practice

Only 28% of respondents indicated that since sustaining their SCI, a health care professional had discussed PFMT with them as a potential treatment option. When asked who had spoken to them about PFMT, 80% of respondents reported a physician, 62% reported a physiotherapist, 42% reported a personal trainer, 32% reported a nurse, and 14% indicated another health care professional, such as an occupational therapist or a rehabilitation trainer. More males than females [χ^2^ (2) = 7.210, *p* = 0.027] and more people with mcSCI compared to those with miSCI [χ^2^ (2) = 22.295, *p* < 0.001] reported that health care professionals had not discussed PFMT with them (Table 3).

**Table 3:** Practice results by sex and degree of motor function.

**Question**	**Response**	**Females** ***n*** **(%)**	**Males** ***n*** **(%)**	**miSCI** ***n*** **(%)**	**mcSCI** ***n*** **(%)**
Did a healthcare professional ever discuss PFMT with you?	Yes	29 (30.5)	13 (22.4)	***40 (36.7)***	***2 (4.5)***
	No	***56 (61.1)***	***45 (77.6)***	***61 (56.0)***	***42 (95.5)***
	Unsure	***8 (8.4)***	***0***	8 (7.3)	0
		**χ^2^ = 7.210**, ***p*** **= 0.027**	**χ^2^ = 22.295**, ***p*** **< 0.001**
Have you tried to do a PFM contraction?	Yes	***68 (71.6)***	***26 (44.8)***	***74 (67.9)***	***20 (45.5)***
	No	***26 (27.4)***	***26 (44.8)***	***31 (28.4)***	***21 (47.7)***
	Unsure	***1 (1.1)***	***6 (10.3)***	4 (3.7)	3 (6.8)
		**χ^2^ = 14.221**, ***p*** **< 0.001**	**χ^2^ = 6.678**, ***p*** **= 0.035**
Have you participated in PFMT?	Yes	23.2	13.8	***27 (24.8)***	***3 (6.8)***
	No	73 (76.8)	48 (82.8)	***80 (73.4)***	***41 (93.2)***
	Unsure	0	2 (3.4)	2 (1.8)	0
		χ^2^ = 5.046, *p* = 0.080	**χ^2^ = 7.512**, ***p*** **= 0.023**
Do you think your PFMT was effective? (*n* = 30)	Yes	15 (68.1)	7 (87.5)	***22 (81.5)***	***0***
	No	1 (4.5)	0	***0***	***1 (33.3)***
	Unsure	6 (27.3)	1 (12.5)	5 (18.5)	2 (66.7)
		χ^2^ = 1.211, *p* = 0.546	**χ^2^ = 14.127**, ***p*** **< 0.001**

*Comparison of Practice between sexes and motor-functions. The bolded statistics are significant. The bolded and italicized comparisons are significant*.

Sixty-one percent of respondents reported that they have tried to do a pelvic floor muscle contraction since sustaining their SCI, with most respondents being female [χ^2^ (2) = 14.221, *p* < 0.001] and with miSCI [χ^2^ (2) = 6.678, *p* = 0.035] (Table 3).

Only 20% (*n* = 30) of respondents reported engaging in a PFMT program, with a significant proportion of them with miSCI [χ^2^ (2) = 7.512, *p* = 0.023]. A majority of those who reported engaging in a PFMT program reported that they felt it was effective (Table 3). Respondents reported that they practiced PFMT by themselves (57%), with a physician (63%), with a physiotherapist (47%), with a personal trainer (33%), or other (23%), such as an app. PFMT programs ranged from sessions being held 1–7 times per week for 2–12 weeks, with or without different types of exercise accessories (Table 4).

**Table 4:** Pelvic floor muscle training characteristics.

**Practice characteristics (*n* = 30)**	**Mean**	**Median**	**Range**
**Times per week**	3.07	2	(1–7)
**Duration of program**			
Number of weeks	6.4375	5.5	(2–12)
Ongoing	*n* = 2		
**Use of exercise accessories**	***n***	**%**	
Biofeedback	17	56.7	
Electrical stimulation	10	33.3	
Visual observation	11	36.7	
Other	10	33.3	

*Details regarding pelvic floor muscle training programs in the sub-set of respondents who reported participation in PFMT (n = 30). Exercise accessories were defined as tools or therapy equipment used as part of the PFMT training program*.

## Discussion

The purpose of this study was to explore knowledge, attitudes, and practices related to PFMT in the SCI population. Generally, people with SCI were aware of PFMT and had favorable attitudes toward it; however, few had actually practiced PFMT. Females were more knowledgeable and generally had stronger belief that they would benefit from PFMT compared to males. More people with miSCI than mcSCI reported that they felt they could benefit from PFMT and had actually participated in a PFMT program.

People with SCI appear to have greater awareness of PFMT when compared to the general population. Surveys of pregnant and post-partum women report awareness of PFMT in the range of 6–56%, compared to 63% of our respondents who indicated knowledge of this type of therapy (21–24). The extent of PFMT knowledge in people with SCI may not be surprising as, following their injury, they may be more aware of the urinary system and associated structures as they work with clinicians to develop individualized bladder management routines (31).

Despite the majority of respondents being aware of PFMT and having favorable attitudes toward this type of therapy, the prevalence of practicing PFMT appears to be low in this population. Most participants indicated that a healthcare worker had never discussed PFMT with them post-injury, which is not surprising given that there are limited studies to date exploring the benefits of PFMT for people with SCI (10–12). Indeed, in international clinical guidelines for treating neurogenic lower urinary tract dysfunction, the use of PFMT is either not included (32), or is mentioned briefly but without clear instruction on how to deliver such a program (33, 34). Confidence and experience in delivering a PFMT is crucial for clinicians to being able to implement these programs. This is demonstrated by a recent survey of obstetric health care workers who indicated that while they were knowledgeable about and had favorable attitudes toward PFMT, they had poor confidence in delivering and evaluating correct performance of PFMT treatment, resulting in fewer of their patients practicing PFMT (35). As our results indicate that people with SCI feel most comfortable discussing PFMT with a health care professional, future work should explore the extent to which practitioners managing the clinical care of people with SCI feel comfortable prescribing PFMT and providing resources on this therapy.

Of the respondents who reported experience previously participating in PFMT, program parameters varied widely, indicating inconsistent PFMT prescription. This is in line with systematic reviews that have found that PFMT programs lack consistency across research studies as well as in clinical practice (36, 37). While there is some evidence to support optimal training loads in able-bodied women with urinary incontinence (38), further research is needed to explore the optimal training parameters for PFMT programs in neurological populations such as those with SCI. However, despite variations in PFMT programs, the majority of respondents perceived that their program as being beneficial.

When compared to female respondents, males were more likely to be unsure about the purpose of PFMT, unsure of where to access PFMT, and less likely to think they could benefit from PFMT. As this therapy is most commonly prescribed to females for post-partum recovery (5), this may be the only context in which males are familiar with PFMT, consequently leading to the presumption that PFMT would not be beneficial to them. Indeed, in our survey, some male respondents specifically answered that they thought PFMT could only be performed by females. However, it is also possible that our survey could have been subject to a sampling bias which could have impacted our findings. If females are more likely to be knowledgeable or interested in PFMT, it is possible we unintentionally attracted more female than male participants by advertising that the survey was about “pelvic floor muscle training.” Indeed, our sample does not reflect the typical male:female distribution of the SCI population; more than two-thirds of our respondents were female, in contrast to the ~30% of the global SCI population being female (27, 28). However, if our higher female participation rates are indicative that females were more likely to engage in this survey topic, this would still support that males may be lacking knowledge on PFMT, suggesting there is need to create sex-specific strategies for education on this topic.

There were no significant differences between people with mcSCI and miSCI with respect to having heard about PFMT. More people with miSCI reported having practiced PFMT. As it is generally assumed that those diagnosed with mcSCI would have no voluntary control over their pelvic floor muscles, it is perhaps unsurprising that clinicians would be less likely to prescribe this type of therapy (14). However, increasing evidence supports that people classified with mcSCI have residual descending motor input to muscles below their injury level (13–17), including the pelvic floor muscles (18). Previous work has also demonstrated that it is possible to train the trunk musculature below the level of injury in people with mcSCI to improve seated balance (15, 17); it remains unclear if the pelvic floor muscles may be similarly trained for improvements in urogenital function. As our results suggest that people with mcSCI are interested in learning more about PFMT and think they could benefit from this type of training, future studies should explore the potential use of PFMT for people with this type of injury.

There are some limitations that should be considered when interpreting the results from this survey. Our online survey was susceptible to false responses as we had to rely on participant's self-screening relative to the eligibility criteria. While we attempted to eliminate these false responses, it is possible that some valid responses may have been erroneously excluded or invalid responses included. However, by using an internet-based survey we were able to reach a large and specific population in a timely and cost-effective manner. Further, respondents, who might have otherwise been hesitant to talk about health-related topics (such as PFMT) face-to-face, were able to participate anonymously (39). This survey did not inquire about respondents' current urogenital health or extent of neurogenic lower urinary tract dysfunction, so we could not evaluate the likelihood that respondents would be candidates for PFMT. However, considering that 80% of the SCI population is reported to experience bladder dysfunction (1), we could assume that a majority of respondents had some degree of neurogenic lower urinary tract symptoms. There may also be sampling bias within the survey in that almost all respondents were from Canada or the United States. Health care systems and the popularity of therapeutic approaches vary globally; the results from this study may only reflect the experiences and perspectives of individuals with SCI who are living in North America.

In conclusion, our results suggest that the majority of people with SCI would like to learn more about PFMT and believe they could benefit from this therapy, but only a small proportion of respondents had actually undertaken a PFMT program. As trained and knowledgeable staff are needed to improve access to PFMT for those with SCI, future studies should explore the KAP of clinicians who would be prescribing and supporting PFMT programs for people with SCI. Differences between males and females regarding baseline knowledge of the pelvic floor muscles and attitudes toward PFMT should be further explored in future studies of the implementation of this therapy in the SCI population.

## Data Availability Statement

The raw data supporting the conclusions of this article will be made available by the authors, without undue reservation.

## Ethics Statement

The studies involving human participants were reviewed and approved by University of British Columbia Clinical Research Ethics Board. The patients/participants provided their written informed consent to participate in this study.

## Author Contributions

MS-K and AW made significant contributions to the study design, data collection, data analysis and interpretation, and drafting and revising this manuscript. WM and TL made significant contributions to developing the idea for the study, study design, interpreting the data, and revising the manuscript. All authors contributed to the article and approved the submitted version.

## Funding

This project was funded by the Canadian Institutes of Health Research (PTJ-166040).

## Conflict of Interest

The authors declare that the research was conducted in the absence of any commercial or financial relationships that could be construed as a potential conflict of interest.

## Publisher's Note

All claims expressed in this article are solely those of the authors and do not necessarily represent those of their affiliated organizations, or those of the publisher, the editors and the reviewers. Any product that may be evaluated in this article, or claim that may be made by its manufacturer, is not guaranteed or endorsed by the publisher.
