# Supplementary material for: Knowledge, Attitudes, and Practice of Pelvic Floor Muscle Training in People With Spinal Cord Injury: A Cross-Sectional Survey
Source: Front Rehabil Sci. 2022 Jun 14;3:893038. doi: 10.3389/fresc.2022.893038 (PMC9397781; doi:10.3389/fresc.2022.893038)
Supplement: Supplementary file 1 [file Data_Sheet_1.docx]

**Online Survey Questions** (Using the online platform Qualtrics)

* indicates response logic

**Captcha:**

Before you proceed to the survey, please complete the captcha below.

**Knowledge:**

| Question | Response | | |
| --- | --- | --- | --- |
|  | Yes | No | Unsure |
| 1. Have you heard of the “pelvic floor muscles” or the “pelvic diaphragm”? |  |  |  |
| 2. Have you heard of “Kegels”? |  |  |  |
| 3. Have you heard of pelvic floor muscle training? |  |  |  |
| *If YES or UNSURE to 3:  4. Where do you think you could go to get pelvic floor muscle training? (If you are unsure, you can write, “I don’t know”.) | [open textbox] | | |
| *If YES or UNSURE to 3:  5. **Who would you go to, to learn about pelvic floor muscle training?** (Please note, the next question will automatically appear after selecting an answer.) | | | |
| a. Another person with a spinal cord injury |  |  |  |
| b. A physician |  |  |  |
| c. A physiotherapist |  |  |  |
| d. A nurse |  |  |  |
| 6a. Is there someone else (not mentioned above) that you would go to, to learn about pelvic floor muscle training? |  |  |  |
| 6b. If “Yes”, who? | [open textbox] | | |
| *If YES or UNSURE to 3:  7. What do you think is pelvic floor muscle training used for? | [open textbox] | | |

**“These are pictures that show the pelvic for males and females. The pelvic floor muscles are located at the bottom.**

**Pelvic floor muscle training can increase the strength of the pelvic floor muscles. Pelvic floor muscle training can be used to decrease urine leakage and improve sexual well-being.**

**Examples of when we would use a pelvic floor muscle contraction include: stopping the flow of urine, preventing the passing of gas, or preventing a bowel movement.”**

**
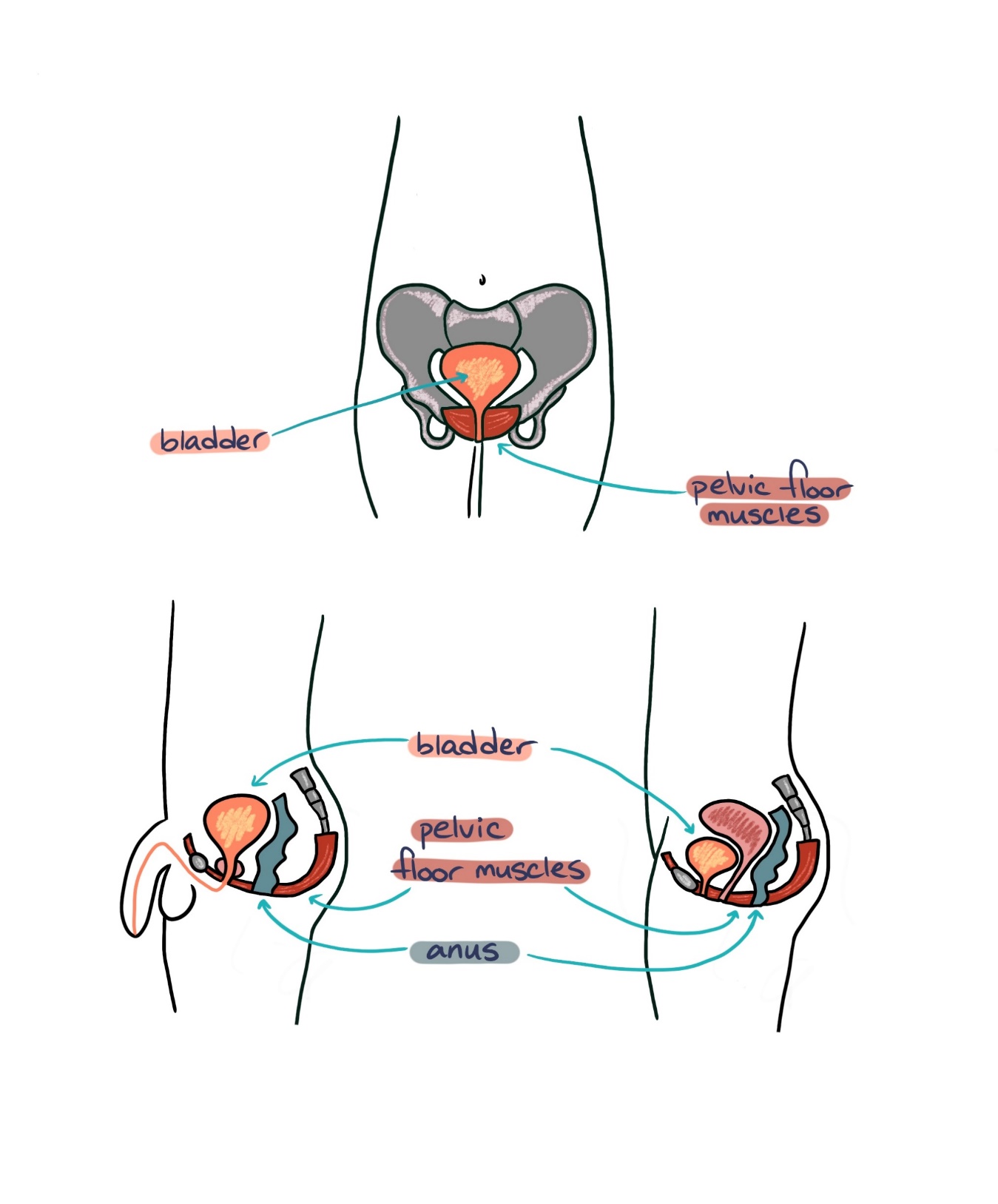
**

**Attitudes:**

**Please indicate how much you either agree or disagree with the following statements:**

| Question | Response | | | | |  |
| --- | --- | --- | --- | --- | --- | --- |
|  | Strongly Agree | Some-  what Agree | Neither agree nor disagree | Some-  what  Disagree | Strongly Disagree | |
| 1. I feel comfortable talking about the pelvic floor muscles to a **physician**. |  |  |  |  |  | |
| 1. I feel comfortable talking about the pelvic floor muscles to a **physiotherapist**. |  |  |  |  |  | |
| 1. I feel comfortable talking about the pelvic floor muscles to a **nurse**. |  |  |  |  |  | |
| 1. I feel comfortable talking about the pelvic floor to **other people with a spinal cord injury**. |  |  |  |  |  | |
| 1. I feel comfortable talking about the pelvic floor muscles to my **friends**. |  |  |  |  |  | |
| 1. I feel comfortable talking about the pelvic floor muscles to my **family**. |  |  |  |  |  | |
| 1. I am confident in my ability to contract my pelvic floor. |  |  |  |  |  | |
| 1. I would like to learn more about pelvic floor muscle training. |  |  |  |  |  | |
| 1. I think I could benefit from a pelvic floor muscle training program. |  |  |  |  |  | |

**Practice:**

| Question | Response | | | | |
| --- | --- | --- | --- | --- | --- |
|  | Yes | No | | Unsure | |
| 1. After your spinal cord injury, did a health care professional or clinician ever discuss pelvic floor muscle training with you as a potential treatment? |  |  | |  | |
| *If YES or UNSURE to 1:  2. **After your spinal cord injury, who discussed pelvic floor muscle training with you?** | | | | | |
| a. A physician |  |  | |  | |
| b. A physiotherapist |  |  | |  | |
| c. A nurse |  |  | |  | |
| d. A personal trainer |  |  | |  | |
| 3a. Is there another professional (not mentioned above) that has discussed pelvic floor muscle training with you? |  |  | |  | |
| *If YES, to 3a:  3b. If “Yes”, who? | [open textbox] | | | | |
| 4. After your spinal cord injury, have you **tried** to do a pelvic floor muscle contraction (Kegel)? |  |  | |  | |
| 5. After your spinal cord injury, have you participated in a pelvic floor muscle training program? (A training program being defined as taking place at least once a week for multiple weeks.) |  |  | |  | |
| *If YES or UNSURE to 5:  6. Are you currently still participating in a pelvic floor muscle training program? |  |  | |  | |
| *If YES or UNSURE to 5:  7. **Thinking about your most recent or current pelvic floor muscle training program, who did you do your training with?** (Please note, the next question will automatically appear after selecting an answer.) | | | | | |
| a. By myself (without the involvement of others) |  |  | |  | |
| b. With a physician |  |  | |  | |
| c. With a nurse |  |  | |  | |
| d. With a physiotherapist |  |  | |  | |
| e. With a personal trainer |  |  | |  | |
| 8a. Have you done pelvic floor muscle training in a scenario not mentioned above? |  |  | |  | |
| *If YES to 8a:  8b. If yes, please explain how and with whom:  (e.g. “I used the app [name of app] with a friend.”) | [open textbox] | | | | |
| *If YES to 5:  9. **If you used any exercise accessories or therapy equipment as part of your program, which one(s) did you use?** (Please note, the next question will automatically appear after selecting an answer.) | | | | | |
| 1. Biofeedback |  | |  | |  |
| 1. Electrical Stimulation |  | |  | |  |
| 1. Visual Observation (e.g. with a mirror) |  | |  | |  |
| 10. Please list any other tools you might have used that were not mentioned above. | [open textbox] | | | | |
| *If YES to 5:  11. How frequent (# of times/week) was your pelvic floor muscle training program? | Drop down option:  1-7 (number of days per week) and I do not remember | | | | |
| *If YES to 5:  12. How many times per day did you do your pelvic floor muscle contractions? | a. Please enter # of times per day below  [open text box]  b. I do not remember | | | | |
| *If YES to 5:  13. How long (# of weeks) was your pelvic floor muscle training program? (If you are currently in a program, indicate how many weeks your program is supposed to last.) | a. Please enter # of weeks per day below  [open text box]  b. I do not remember | | | | |
| *If YES to 5:  *SKIP if YES to 6:  14. Why did you stop the pelvic floor muscle training program? | 1. I completed the pelvic floor muscle training program. 2. I stopped the pelvic floor muscle training program because: [open textbox] | | | | |
| *If YES to 5:  15. Do you think your pelvic floor muscle training program was/is effective? | Yes | No | | Unsure | |
|  | “please explain” beside each answer and an open text box beside each answer | | | | |

**Demographics:**

1. **When is your date of birth?**

Please select: [month] [year]

1. **Sex:**
2. Female
3. Male
4. Intersex
5. Prefer not to disclose
6. **Do you identify as a woman, man or non-binary person?**
7. Woman
8. Man
9. Nonbinary
10. Prefer not to disclose
11. **When did you have your spinal cord injury?**

If known, please select**:** [month] [year]

1. **What was the cause of your spinal cord injury?**
2. External (e.g. motor vehicle accident, fall, violence, sports, etc…)
3. Other (e.g. tumour, infection, congenital, disease, etc…)
4. **What is the level of your spinal cord injury? (Please include vertebral level if known.)**
5. Cervical [drop down with options: C1, C2, C3, C4, C5, C6, C7, C8]
6. Thoracic [drop down with options: T1, T2, T3, T4, T5, T6, T7, T8, T9, T10, T11, T12]
7. Lumbar [drop down with options: L1, L2, L3, L4, L5]
8. Sacral [S1, S2, S3, S4, S5]
9. **Muscle Function (please select one option below):**
10. I have some ability to activate the muscles below my level of injury
11. I am not able to activate any of the muscles below my level of injury
12. **Which country do you currently live in?**
13. Canada
14. United States of America
15. **Other country (please select):**

[drop down list of countries]

*If selected UNITED STATES OF AMERICA in 11:

1. **Which state do you currently live in?**

[drop down list of American states]

*If selected CANADA in 11:

1. **Which province/territory do you currently live in?**

[drop down list of Canadian provinces and territories]

1. **How would you best describe your location of residence?**
2. City
3. Suburban
4. Rural
5. **What is your highest level of education completed?**
6. Less than high school
7. High school
8. College or trade school
9. Post-Graduate Degree
